# Extended phenotype of phytoplasmas in eukaryotic systems: mechanisms and ecological implications

**DOI:** 10.3389/fpls.2026.1774394

**Published:** 2026-04-10

**Authors:** Eswaran Priyadharshini, Maddi Sandhya, Theerthagiri Anand, Mahalingam Angamuthu, Marimuthu Murugan, Nagendran Tharmalingam, Govindasamy Senthilraja

**Affiliations:** 1 Department of Plant Pathology, Centre for Plant Protection Studies, Tamil Nadu Agricultural University, Coimbatore, Tamil Nadu, India; 2 Regional Research Station, Tamil Nadu Agricultural University, Vriddhachalam, Tamil Nadu, India; 3 Department of Agricultural Entomology, Centre for Plant Protection Studies, Tamil Nadu Agricultural University, Coimbatore, Tamil Nadu, India; 4 Department of Medicine, Houston Methodist Research Institute, Houston, TX, United States

**Keywords:** ecosystem, effectors, extended phenotypes, management, mechanisms, phytoplasmas

## Abstract

Manipulation of host behavior is believed to result from parasitic genes influencing the host’s genes or its environment. This adaptive strategy, known as an extended phenotype, boosts the fitness and adaptation of pathogens. Numerous examples of extended phenotypes exist in nature, including zombie ants, fearless mice, brood parasitism in cuckoos, and zombie spiders, some of which are discussed in this review. In certain cases, parasitic pathogens cause morphological changes in their hosts that directly benefit the parasites by enhancing their adaptive fitness. Notably, plant pathogens, such as phytoplasmas, display extended phenotypes on hosts and insect vectors through the secretion of effector proteins like SAP54, PHYL1, SAP11, SAP05, TENGU, SWP1, SJP1, SJP2, Zaofeng3 (SJP3), SJP39, and Zaofeng6. These effector proteins are key factors in producing phenotypic changes in host plants that increase plant attractiveness to leafhopper vectors by targeting and degrading key transcription factors and developmental regulators. This process aligns with the concept of extended phenotype, as it significantly improves the adaptive fitness of phytoplasmas. This review explores the extended phenotype of phytoplasmas on dual eukaryotic hosts, focusing on effector proteins, their mechanisms, and modern strategies to counteract them.

## Highlights

- Extended phenotypes influence the host behaviour and traits in ways that enhance pathogen fitness.
- Apart from animal ecosystems, plant pathogens such as phytoplasmas also exhibit extended phenotypes on their hosts through effector proteins.
- Effector proteins secreted by phytoplasmas such as, SAP54, PHYL1, SAP11, SAP05, TENGU, SWP1, SJP1, SJP2, Zaofeng3 (SJP3), SJP39, and Zaofeng6 induce phenotypic changes in plant hosts.
- Phytoplasma effector proteins also influence vector attraction and reproduction, providing insights for sustainable management strategies.

## 1 Introduction

The most fascinating adaptations in nature are those shown by parasites that modify their host’s behaviour in adaptive ways (Hughes, 2013). The influence of genes that extends beyond the individual into the environment is called an extended phenotype (EP) (Dawkins, 1982; Huang and Hogenhout, 2022). Parasite genes impact the host’s phenotype to improve the parasites’ survival and reproduction within the host (Orlovskis and Hogenhout, 2016). For example, severe limb deformities caused by the trematodes *Ribeiroia* in frogs hinder the frogs’ movement, increasing their risk of being eaten by birds. But this is crucial for spreading the trematodes into the environment. Here, the trematode genes influence the hosts’ phenotype by manipulating the frog hosts for their own benefit (Johnson et al., 2004). However, the molecular mechanisms behind this process are still not fully understood.

Microorganisms like phytoplasmas that infect plant hosts often manipulate the host’s behaviour to help them colonize the plant (MacLean et al., 2014). Some plant pathogens, such as viruses, require insect vectors to spread. These pathogens cause physiological changes in their plant hosts and modify insect feeding behaviour to favour feeding on infected plants, which increases the likelihood of transmitting the pathogen (Ingwell et al., 2012; Heck, 2018). Phytoplasmas are also part of this group, as they produce a virulence factor (SAP11 effector) that modulates plant physiological processes to attract insect vectors toward infected plants (Sugio et al., 2011a; Tomkins et al., 2018).

Phytoplasmas are unculturable, plant-pathogenic bacteria belonging to the class Mollicutes. These vascular pathogens are specifically confined to the cytoplasm of sieve cells in the phloem, the plant tissue responsible for transporting nutrients and carbohydrates to sink tissues (Sugio et al., 2011a). Phytoplasmas spread throughout the plant via pores in the sieve plates. They are mainly transmitted by sap-feeding leafhopper vectors, and occasionally by plant hoppers and psyllid vectors that feed on the sieve cells of the phloem, where phytoplasmas reside in the host plant (Sugio et al., 2011a, Sugio et al., 2011b; MacLean et al., 2014; Huang et al., 2021). Leafhoppers are sap-feeding insects that feed on nutritious phloem regions and acquire pathogens. Inside the insect’s intestinal lumen, phytoplasmas replicate within epithelial and nearby muscle cells lining the hemolymph. The bacteria are then released into the hemolymph, from which they infect other parts of the insect. They accumulate in vacuoles within the salivary glands and are transmitted during feeding into a healthy host, completing their cycle (Sugio et al., 2011b).

Pathogens secrete effector proteins that act as pathogenicity factors, enhancing their reproduction and survival within host plants. Similarly, phytoplasmas produce various effector proteins, such as SAPs (secreted Aster yellows proteins), SWP1, and Zaofeng6. These proteins modify plant physiological processes, leading to different symptoms in infected hosts (Sugio et al., 2011a; Huang et al., 2021). Some well-documented symptoms caused by these effectors include: 1) SAP54 induces phyllody, 2) SAP11 causes witches broom, 3) SAP05 promotes witches broom and delays senescence (Huang and Hogenhout, 2022), 4) PHYL1 causes phyllody (Maejima et al., 2014), 5) Zaofeng3 triggers phyllody (Chen et al., 2024a), 6) Zaofeng6 (Yang et al., 2022), SWP1 (Wang et al., 2018a), SJP1, and SJP2 (Zhou et al., 2021) lead to witches broom, 7) SJP39 results in stunted growth (Yang et al., 2024) and 8) SRP1 causes leaf yellowing symptoms (Zhang et al., 2024). These effectors not only induce symptoms in hosts but also influence insect vector behaviour by manipulating them to feed on and colonize infected plants (MacLean et al., 2014; Tomkins et al., 2018). Consequently, phytoplasmas extend their phenotype across kingdoms to affect both insect and plant hosts. This review explores in depth the role of phytoplasma effector proteins in modulating plant and insect physiology to improve pathogen fitness.

## 2 The EP concept

In biology, genotype refers to an organism’s genetic makeup, while phenotype describes its morphology and behaviour (Pierce, 2020; Griffiths et al., 2019). However, a gap exists between genotype and phenotype concerning the interaction between a plant and the organisms it hosts. The process by which the genotype of one organism influences the phenotype of another organism or host is called extended phenotype (EP). EPs result from interactions between the host and parasite genomes (Lambrechts et al., 2006). Richard Dawkins first introduced the concept of EPs in his book “The Extended Phenotype” (1982) (Dawkins, 1982). It is also known as the “selfish gene” concept. He explains that organisms tend to alter their environment to their advantage rather than passively living within it (Turner, 2004). Dawkins also states that EP arises through the natural selection of genes in the environment (Hughes, 2013). His initial outline of EP describes three different forms of EP (Form I, II and III) that are still recognized today. In 2018, a fourth form (Form IV) has been introduced involving complex ecosystems as a unifying action of I and II (**Table 1**) (Hunter, 2018).

**Table 1:** Forms of extended phenotype (Dawkins, 1982).

Form	No. of species involved	Mechanism	Examples	References
I	Single species	Niche construction / animal architecture	Beaver dams	Puttock et al. (2021)
			Termite mounds and fungal gardens	Enagbonma and Babalola (2019)
II	Involving two or three species	Manipulation of host behaviour by parasites by direct interaction or alteration of gene expression	Suicidal crickets and grasshoppers infected with hair worms	Libersat et al. (2009)
			Zombie ants infected by fungus, *Ophiocordyceps unilateralis*	De Bekker et al. (2015)
			Giant turtle ants infected by tetradonematid nematodes	Hubregtse (2019)
			Zombie spiders infected by the fungus, *Gibellula* sp.	Saltamachia (2022); Mendes-Pereira et al. (2023)
III	Involving 2 species	Action at a distance	Brood parasitism by cuckoo birds	Hughes (2013); Hunter (2018)
IV	Complex ecosystems (unified action of I & II forms)	Involves both architecture and the microorganisms dwelling on the hosts	Soil ecosystem involving plants and diverse microorganisms	Hunter (2018)

Darwin’s quote (1859) (Darwin, 1859), “From so simple beginnings, endless forms most beautiful and most wonderful have been and are being evolved,” highlights the continuous evolution of life (Hughes, 2013). Fossils provide crucial evidence for understanding the history and evolution of organisms, including the behaviour of higher animals (Plummer, 2004; Prothero, 2017). Interestingly, extended phenotypes (EPs) can also be traced in fossils, such as the “death grip” behaviour of ants infected by *Ophiocordyceps unilateralis* preserved in a 48-million-year-old fossil leaf from Messel, Germany (Hughes et al., 2011). Additional examples for fossilized EP are summarized in **Supplementary Table 1**. Despite the frequent occurrence of EPs in nature, the molecular mechanisms underlying many of them remain poorly understood, and several well-established EPs are listed in **Supplementary Table 2**.

## 3 Extended phenotypes of phytoplasmas on plants and insects – a three-way interaction

**Figure 1:**
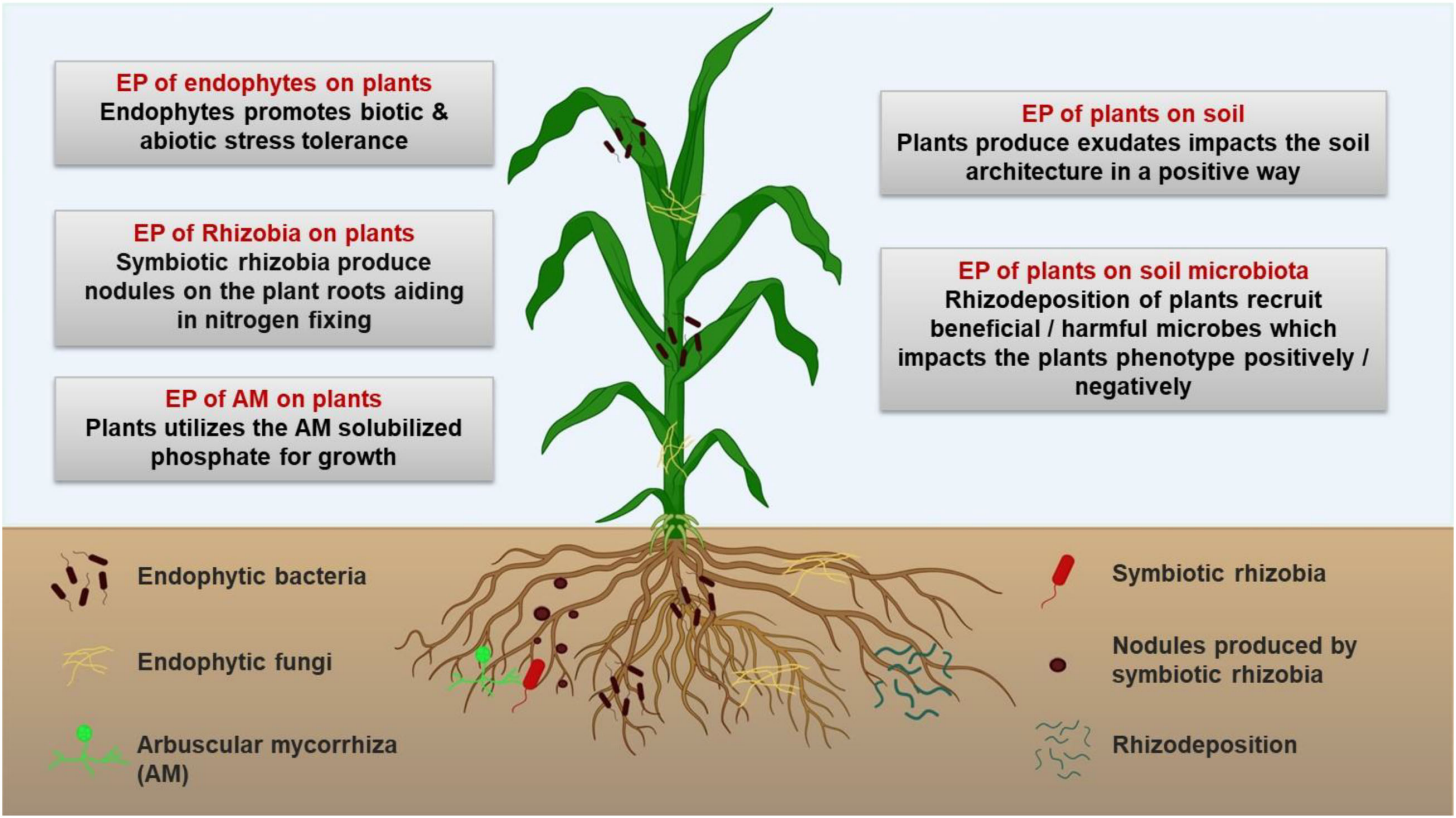
Extended phenotypes (EPs) developed in the context of the plant-microbe ecosystem. This image was created using https://BioRender.com.

Plant-microbe interactions are a key research area because of their effects on plant ecosystems. Microorganisms, especially endophytes and epiphytes, promote plant growth by enhancing plant fitness and stress tolerance (Hawkes et al., 2021; Ruiz-González and Vicente, 2022). The plant microbiome varies with genotype, influencing the rhizosphere microbiota and causing several changes in the rhizosphere (Wagner, 2021). Therefore, the rhizosphere (soil and microbiota) can be seen as an extended root phenotype, and vice versa (**Figure 1**) (de La Fuente Cantó et al., 2020).

However, microbes can also cause harmful phenotypes, such as galls produced by the actinomycete *Rhodococcus faciens* through the production of cytokinins (Giron and Glevarec, 2014), which can impact host adaptation, spread, and survival in plants (Thines and Kamoun, 2010). One such adaptive organism is the phytoplasma. They adapt within their hosts by changing plant morphology, as well as affecting longevity, reproductive rates, and the behaviour of insect vectors (Sugio et al., 2011a). They colonize phloem sieve cells and cause symptoms including witches’ broom (stem proliferation), phyllody (modification of flowers into small leaf-like structures), floral virescence (development of green-colored flowers), yellowing, leaf size reduction, dwarfing, purple tops, brittle crinkled leaves, phloem necrosis, rosetting, stunting, and more (Bertaccini and Duduk, 2009; Orlovskis and Hogenhout, 2016; Ermacora and Osler, 2019; Wang et al., 2024a).

Here we focus on the phytoplasma-induced changes in their plant and insect hosts. Like other bacterial pathogens, phytoplasmas also secrete effector proteins that are essential for altering host cell structure and function to enable infection. Effectors can be seen as ‘the parasitic genes that change the host phenotype and behavior.’ Because these effectors are products of the parasite genome, phytoplasma effectors serve as an example of the concept of EP (Hogenhout et al., 2009). The plants infected by phytoplasmas are called ‘zombie plants’ due to the effector-driven manipulation of the plant hosts (Huang and Hogenhout, 2022; MacLean et al., 2014; Rümpler et al., 2015; Orlovskis et al., 2025).

In brief, phytoplasma is a master manipulator that secretes effectors to infect the host systemically, promoting pathogenesis and altering the host phenotype (Orlovskis et al., 2025). The effectors also disrupt the host signaling pathways, leading to the attraction of viruliferous insect vectors toward infected plants. The effects of phytoplasma effectors on plant and insect hosts are discussed below (**Table 2**).

**Table 2:** Extended phenotype of phytoplasmas.

Host	Effectors	Targets	Extended phenotype	Group of phytoplasma	Disease^a^	References
Model plant – *Arabidopsis thaliana* transgenic for the diverse effectors	SAP 54	MADS-box protein responsible for floral development	Conversion of floral parts into leafy structures (phyllody)	*Ca.* P. asteris	AY-WB	MacLean et al. (2011)
		MADS-box transcription factor SVP	Phyllody and attraction of female leafhopper vectors			Orlovskis et al. (2025)
	PHYL1	MADS domain proteins, SEP3, AP1 and CAULIFLOWER (CAL)	Phyllody	*Ca.* P. asteris	OY	Iwabuchi et al. (2020)
				*Ca.* P. australasiaticum	PnWB	
				*Ca.* P. ziziphi	JWB	Xue et al. (2024)
	PHYL1	Upregulates the negative flowering regulator gene SVP	Leafy flower phenotypes (Phyllody)	*Ca.* P. australasiaticum	PnWB	Yang et al. (2015)
	SAP11	CIN-TCP TFs responsible for growth and development of plants	Witches’ broom and stem proliferation Additionally, prevents growth of male & female inflorescence in AY-WB & MBSP respectively	*Ca.* P. asteris	AY-WB MBSP	Sugio et al. (2011a)
				*Ca.* P. australasiaticum	WBDL	Al-Subhi et al. (2021)
				*Ca.* P. mali	AP	Tan et al. (2016); Mittelberger et al. (2022)
			Crinkled and reduced leaf size, more axillary branches, modifications in root morphology, smaller and crinkle siliques	*Ca.* P. solani	Stolbur disease	Drcelic et al. (2024)
	SJP1 & SJP2	ZjBRC1 promoting the auxin accumulation	Witches’ broom and lateral bud growth	*Ca.* P. ziziphi	JWB	Zhou et al. (2021)
	SJP3/Zaofeng3	Several MADS-box TF genes - crucial for floral organ identity and flowering time	Phyllody, witches’ broom, malformed floral organs and dwarfism			Chen et al. (2024a)
	SAP05	SPL and GATA TFs	Delayed plant senescence and witches’ broom	*Ca.* P. asteris	AY-WB	Huang et al. (2021)
	TENGU	ARF6 and ARF8 genes by exhibiting misregulation of auxin and JA signaling pathways	Stunting, witches’ broom and sterility	*Ca.* P. asteris	OY	Minato et al. (2014)
	Zaofeng6	ZjTCP7 leading to the downregulation of strigolactone signalling pathway	Witches’ broom	*Ca.* P. ziziphi	JWB	Chen et al. (2022)
	SJP39	Stabilizes ZjbHLH87 and manipulates GA signalling pathway	Stunted growth and other growth defects	*Ca.* P. ziziphi	JWB	Yang et al. (2024)
Model plant- *Nicotiana benthamiana* transgenic for the diverse effectors	SAP11	NbOMT1 encoding O-methyl transferase, leading to the suppression of IBMP volatile	Change in aroma phenotype	*Ca.* P. mali	AP	Tan et al. (2016)
	PHYL1	RAD23 and MTFs	Phyllody	*Ca.* P. oryzae	RYD	Suzuki et al. (2024)
	SWP1	TCP18 (BRC1) which is responsible for arresting bud development	Witches’ broom	*Ca.* P. tritici	WBD	Wang et al. (2018b)
	SJP3	Accumulation of ZjSVP3 involved in determining flowering time	Pistil reversion	*Ca.* P. ziziphi	JWB	Deng et al. (2024)
	SAP54 homolog, RY348	MADS-box transcription factors MADS1 and MADS15	Pollen sterility	*Ca.* P. oryzae	RYD	Zhang et al. (2025a)
	SAP54 homolog, RY378	Influences the auxin and strigolactone signalling pathways	Increased tillering			
Rice	SRP1	GS2 reduces the accumulation of chlorophyll precursors	Leaf yellowing and increased attraction of leafhopper vectors	*Ca.* P. oryzae	ROLP	Zhang et al. (2024)
Zig-zag leafhopper (*Recilia dorsalis*)	SRP1	Serine protease 2 (SP2) inhibits SP2-mediated melanization in insects	Inhibited melanization, an innate immune reaction in insects	*Ca.* P. oryzae	ROLP	Zhang et al. (2025b)
Insect vector -Leafhopper (*Macrosteles quadrilineatus*)	SAP54	–	Increases insects colonization on plant hosts and helps in reproduction	*Ca.* P. asteris	AY-WB	Oshima et al. (2023)
	SAP11	CIN-TCP TFs and LOX2		*Ca.* P. asteris	AY-WB	Sugio et al. (2011a)
				*Ca.* P. mali	AP	Tan et al. (2016)
	SAP05	–		*Ca.* P. asteris	AY-WB	Huang and Hogenhout (2022)
Maize leafhopper (*Dalbulus maidis*)	SAP11	–	Non-host attraction to AY phytoplasma	*Ca.* P. asteris	AY-WB	Sugio et al. (2011b)

a AY-WB, Aster yellow witches broom; OY, Onion yellows; PnWB, Peanut witches’ broom; JWB, Jujube witches’ broom; MBSP, Maize bushy stunt phytoplasma; WBDL, Witches’ broom disease of lime; AP, Apple proliferation; RYD, Rice yellow dwarf; WBD, Wheat blue dwarf phytoplasma; ROLP, Rice orange leaf phytoplasma.

### 3.1 SAP54 effector-related host manipulation

**Figure 2:**
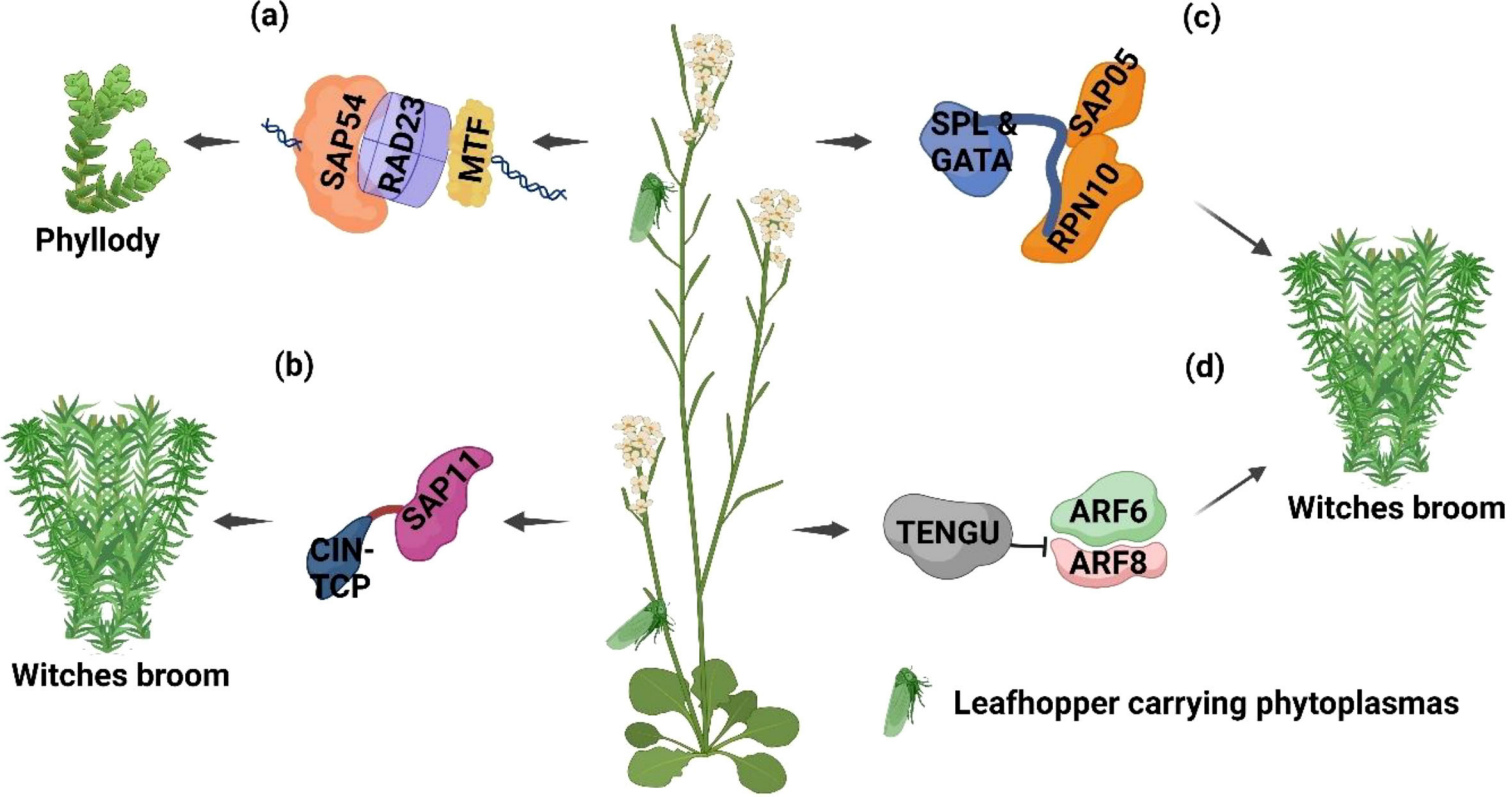
Extended phenotypes (EPs) developed due to effectors released by phytoplasmas on plants. **(a)** SAP54 binds to and degrades MTFs by interacting with RAD23 family proteins, leading to phyllody. **(b)** SAP11 effectors disrupt CIN-TCP transcription factors by binding to them, resulting in witches’ broom and stem proliferation phenotypes. **(c)** SAP05 degrades TFs from the SPL and GATA families, causing witches’ broom symptoms. **(d)** TENGU suppresses the expression of ARF6 and ARF8 genes, also producing witches’ broom symptoms. This image was created using https://BioRender.com.

Previously, the genome of the Aster yellows phytoplasma, *Ca.* P. asteris, was thoroughly analyzed, leading to the identification of 56 secreted AY-WB proteins (SAPs) as effector genes (Bai et al., 2009; Sugio et al., 2011b; Tomkins et al., 2018; Capdevielle, 2019; Pecher et al., 2019). Extensive research has uncovered the mechanisms of these effectors (MacLean et al., 2011, MacLean et al., 2014), which are discussed below. Phyllody is a prominent symptom caused by phytoplasmas in plant hosts. The phytoplasma effector protein SAP54 was found to be responsible for inducing this symptom (Orlovskis and Hogenhout, 2016; MacLean et al., 2011, MacLean et al., 2014). The MADS domain transcription factors (MTFs; MINICHROMOSOME MAINTENANCE FACTOR 1, AGAMOUS-like, DEFICIENS, serum response factor) and key regulators SEPALLATA3 (SEP3) and APETALA1 (AP1) are essential for flower development in plants (Orlovskis and Hogenhout, 2016). The phytoplasma effector SAP54 binds to and degrades MTFs by interacting with the RADIATION SENSITIVE23 (RAD23) family of proteasome shuttle proteins (**Figure 2a**). This mechanism was confirmed in Arabidopsis wild type Col-I, where RAD23 Arabidopsis (wild type Col-I) mutant lines failed to convert flowers into leaf-like structures during phytoplasma infection (Huang et al., 2021; MacLean et al., 2011, MacLean et al., 2014; Sugio et al., 2011b; Tomkins et al., 2018).

**Figure 3:**
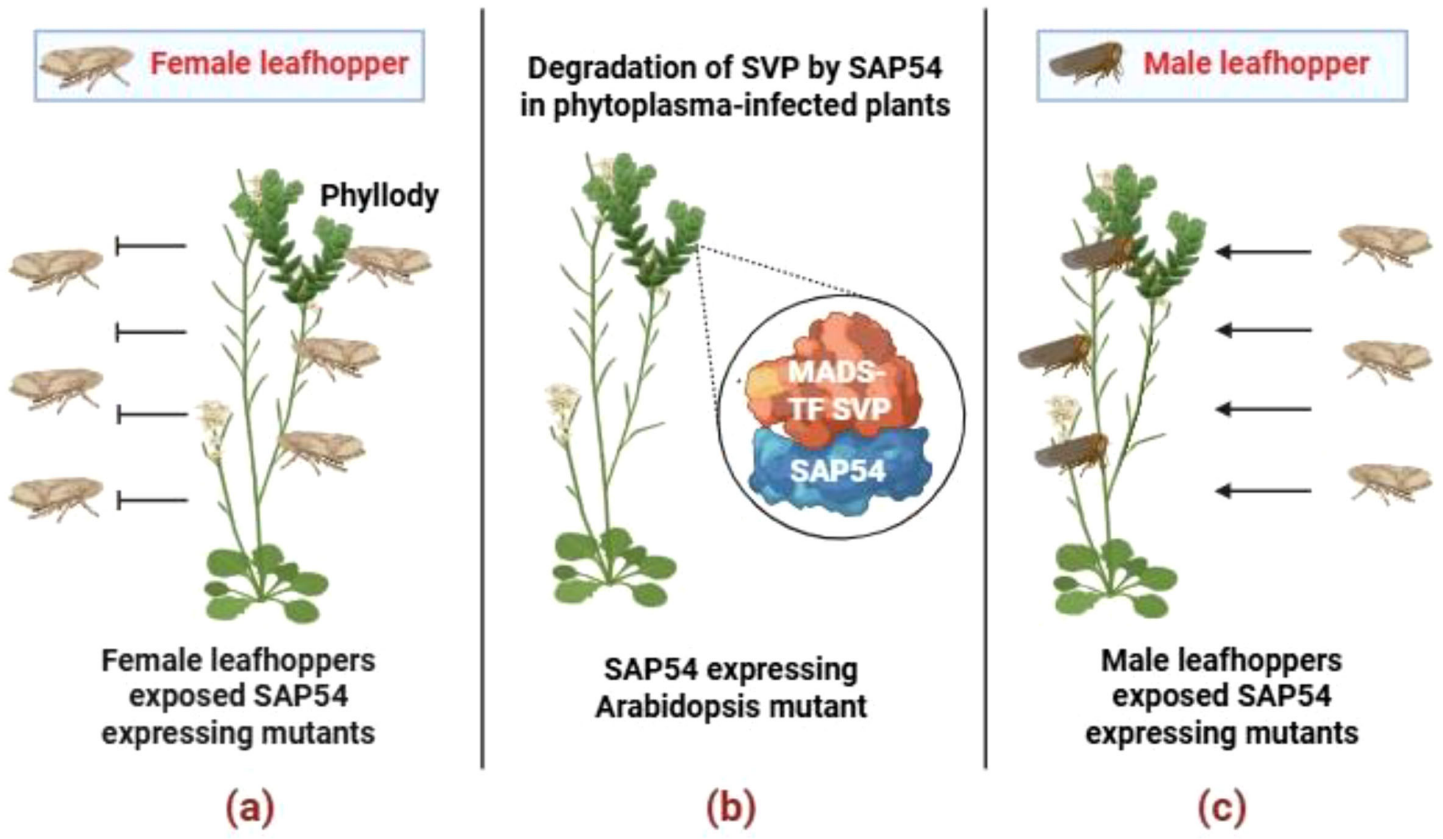
Influence of SAP54 on plant (*A. thaliana*) and insect (*M. quadrilineatus*) hosts, suggesting SAP54 acts as a molecular matchmaker. **(a)** Mutants expressing SAP54 and colonized by female leafhoppers repel other females from colonizing. **(b)** SAP54 degrades the MADS-box transcription factor SHORT VEGETATIVE PHASE (SVP) and induces phyllody. **(c)** Mutants expressing SAP54, when exposed to male leafhoppers, downregulate biotic stress responses by degrading MADS-box TF SVP inducing phyllody and thereby attracting female leafhoppers, indicating that both SAP54 and males are necessary for female colonization. This image was created using https://BioRender.com.

Intriguingly, SAP54 also enhances leafhopper colonization of infected hosts. Leafhopper (*Macrosteles quadrilineatus*) shows increased reproductive preference on phytoplasma infected plants and SAP54 expressing transgenic *Arabidopsis* plants, while the MTF mutant plants in the absence of SAP54 did not attract the leafhoppers (Orlovskis and Hogenhout, 2016). Also, the plants with SAP54 expressed leaf-like flowers (phyllody) attracted more leafhoppers towards AYWB infected plants through a RAD23 dependent manner (MacLean et al., 2014; Tomkins et al., 2018). Collectively, these results establish that SAP54 is essential for phyllody formation, vector attraction and vector-mediated transmission. Recently, Orlovskis et al. (2025) demonstrated a novel mechanism exhibited by SAP54, which is secreted by AY-WB phytoplasma in the attraction of *M. quadrilineatus*. They showed that SAP54 promotes the degradation of the MADS-box transcription factor (TF) SHORT VEGETATIVE PHASE (SVP) and enhances the attraction and colonization of female leafhopper vectors toward male-exposed plants. SVP mutants exposed to males mimic female preference for the SAP54-expressing plants that are male-exposed. Therefore, the SAP54 effector acts as a molecular matchmaker, targeting SVP to downregulate biotic stress responses in male-exposed plants and increase leafhopper susceptibility, thereby promoting the replication of female vectors on male-exposed plants (**Figure 3**). Thus, SAP54 exemplifies an extended phenotype, as phytoplasma genes alter host plant development and insect vector behaviour to promote pathogen fitness.

### 3.2 SAP54 homologs of phytoplasmas

PHYL1 (phyllody 1), a SAP54 homolog identified from *Ca.* P. asteris, onion yellows phytoplasma (OY-W; OY strain; wild type line), has also been shown to induce phyllody in the model plant *Arabidopsis thaliana*. It was found that PHYL1 interacts with and degrades the MADS domain proteins, SEP3, AP1, and CAULIFLOWER (CAL), inhibiting their functions and leading to extended phyllody phenotypes in infected plants (Maejima et al., 2014; Liao et al., 2019; Kitazawa et al., 2022). *Ca.* P. australasiaticum also secretes the PHYL1 effector. In this case, the effector upregulates the negative flowering regulator gene SVP and causes leafy flower phenotypes in Arabidopsis plants (Yang et al., 2015).

Similarly, the secreted jujube protein 3 (SJP3), also known as Zaofeng3 (its name in Chinese – ‘Zao’ means jujube and ‘feng’ means uncontrollable due to its severity), is a SAP54 homolog that has been shown to induce phyllody, witches’ broom, malformed floral organs, and dwarfism in Arabidopsis plants. These phenotypic changes result from disrupting the expression of several MADS-box TF genes, which are crucial for floral organ identity and flowering time (Deng et al., 2021; Chen et al., 2024a). However, the specific function and mechanism of SJP3 in symptom development remain unclear (Chen et al., 2024a). Additionally, the effector is involved in pistil reversion by interacting with the MADS-box protein SHORT VEGETATIVE PHASE 3 (ZjSVP3) and promoting its accumulation. Since SVP genes are essential for regulating flowering time, this interaction inhibits their degradation and interferes with pistil development in hosts (Deng et al., 2024).

Two key SAP54 homologs, RY348 and RY378, have been reported to be involved in the pathogenesis of *Ca.* P. oryzae, which causes rice yellow dwarf (RYD). RY348 causes pollen sterility by degrading the MADS-box transcription factors MADS1 and MADS15, while RY378 influences the auxin and strigolactone signaling pathways, leading to increased tillering (Zhang et al., 2025a). Together, these findings highlight an extended phenotype (EP) in which SAP54 homologs such as PHYL1, SJP3, RY348, and RY378 manipulate host floral regulatory networks and hormone pathways to alter plant development and promote phytoplasma fitness.

### 3.3 SAP11 and the EP

**Figure 4:**
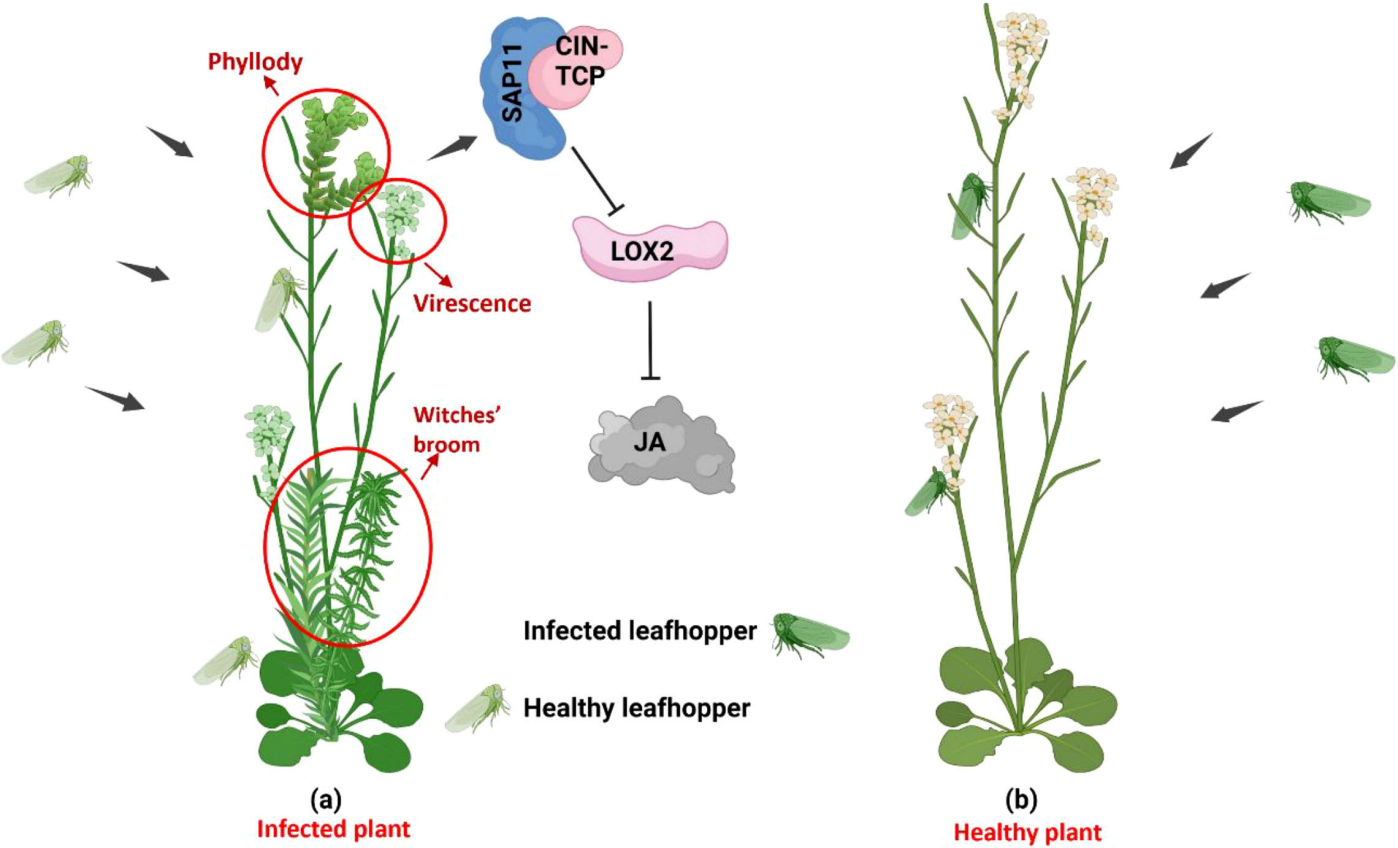
Effects of extended phenotypes (EPs) of phytoplasmas on insects. **(a)** SAP11 destabilizes the CIN-TCP transcription factors, which causes a decrease in LOX2 enzyme levels (a key regulator in jasmonic acid (JA) production). Since JA is not produced in infected plants, more healthy leafhoppers are attracted to phytoplasma-infected hosts. **(b)** Normal feeding of a infected leafhopper on healthy plants. This image was created using https://BioRender.com.

Phytoplasma infection can cause stunting and excessive axillary branching, resulting in a witches’ broom phenotype in infected plants. Consistent with this, Arabidopsis plants expressing SAP11 phytoplasma effector developed witches’ broom symptoms. SAP11 effectors inhibit CIN-associated TEOSINTE BRANCHED1, CYCLOIDEA, PROLIFERATING CELL FACTORS 1 and 2 (TCP) transcription factors (TFs) by binding to them, contributing to the witches’ broom and stem proliferation phenotypes (**Figure 2b**) (Sugio et al., 2014, Sugio et al., 2011a; Huang et al., 2021; Huang and Hogenhout, 2022). The destabilization of CIN-TCP TFs suppresses LIPOXYGENASE2 (LOX2) gene expression, which encodes a key enzyme in jasmonic acid (JA) biosynthesis—a phytohormone vital for insect resistance in plants (**Figure 4**). This mechanism promotes *M. quadrilineatus* vector colonization of aster yellows infected China aster (*Callistephus chinensis* Nees) and SAP11-expressing *Arabidopsis* plants, increasing their survival and reproduction rates on phytoplasma-infected plants (Sugio et al., 2011a, Sugio et al., 2011b; Tomkins et al., 2018).

The AY-WB infected plants (*Nicotiana benthamiana*, *A. thaliana*, lettuce, china aster and maize) attracted not only *M. quadrilineatus* but also *Dalbulus maidis*, a maize specialist leafhopper. *D. maidis*, which usually cannot survive or feed on non-maize species, surprisingly exhibited increased survival and reproduction on these diverse AY-WB infected hosts. However, *D. maidis* failed to acquire and transmit the pathogen (Purcell, 1988; Heather and Hogenhout, 2007; Sugio et al., 2011b). The mechanism by which phytoplasmas manipulate and suppress non-host resistance to *D. maidis* remains unclear, and this non-host attraction is hypothesized to be the EP of the AY-WB phytoplasma, potentially mediated by the effector SAP11 (Sugio et al., 2011b).

Additionally, SAP11, produced by *Ca.* P. mali, alters the aroma profile of infected *N. benthamiana* plants. The O-methyltransferase encoded by NboMT1 normally produces the volatile organic compound 3-isobutyl-2-methoxypyrazine (IBMP). Functional genomic analysis showed that NboMT1 is significantly downregulated in plants with the SAP11 transgene (Tan et al., 2016). These findings suggest that phytoplasmas affect plant and aroma traits by disrupting secondary metabolism pathways via the SAP11 effector, which contributes to symptom development and facilitates pathogen spread.

### 3.4 SAP11-like effectors

The SAP11 homolog, SAP11_WBDL_, found in *Ca.* P. australasiaticum—the pathogen responsible for witches’ broom disease of lime—interacts with TCP transcription factors and causes witches’ broom symptoms in lime and transgenic *A. thaliana*. Additionally, branches exhibiting these symptoms on Omani lime trees had higher SAP11_WBDL_ levels and attracted more leafhopper vectors (*Hishimonus phycitis*) compared to healthy, asymptomatic branches (Al-Subhi et al., 2021).

The recently characterized SAP11-like effector Zaofeng6, produced by *Ca.* P. ziziphi, interacts with and decreases the expression of ZjTCP7 (*Ziziphus jujuba* TCP7). This results in the down-regulation of genes involved in the strigolactone signaling pathway, leading to witches’ broom symptoms in the infected *N. benthamiana* plants (Chen et al., 2022; Yang et al., 2022).

SJP1 and SJP2 are SAP11-like effectors secreted by the jujube witches’ broom phytoplasma, have been confirmed to trigger witches’ broom symptoms. Their coiled-coil domains target the CYC/TB1 - TCP TFZjBRC1 in the nucleus. Normally, ZjBRC1 represses the promoters of auxin efflux carriers ZjPIN1c/3, leading to auxin buildup in jujube calli. During infection, these effectors inhibit ZjBRC1 and activate ZjPIN1c/3, encouraging lateral bud growth and resulting in witches’ broom symptoms (Zhou et al., 2021; Ma et al., 2023, Ma et al., 2024).

These findings illustrate an extended phenotype (EP) in which the phytoplasma effector SAP11 and SAP11-like effectors manipulates host transcription factors, hormone signaling, and volatile profiles to alter plant architecture and vector behaviour, thereby enhancing pathogen transmission.

### 3.5 Secreted JWB protein 39

The *Ca.* P. ziziphi effector SJP39 influences symptom development by modulating gibberellic acid (GA) signalling in the host. It specifically stabilizes ZjbHLH87 (*Ziziphus jujuba* basic helix-loop-helix 87), a negative regulator of the GA pathway, thereby amplifying this regulation and resulting in stunted growth and additional growth defects in SJP39- and ZjbHLH87- overexpressing transgenic *A. thaliana* and jujube plants (Yang et al., 2024).

### 3.6 SAP05 mediated host modification

SAP05, a significant effector secreted by *Ca.* P. asteris, targets transcription factors from the SPL (SQUAMOSA-promoter binding protein-like) and GATA families by hijacking the plant’s ubiquitin receptor RPN10 (**Figure 2c**). This interference results in delayed leaf senescence, witches’ broom symptoms in infected Arabidopsis, and suppression of the plant’s defenses against *M. quadrilineatus* vectors. Nonetheless, the exact mechanism by which SAP05 enhances host susceptibility to leafhopper vectors remains unknown (Huang et al., 2021; Huang and Hogenhout, 2022; Zhang et al., 2023).

### 3.7 TENGU and the plant hosts

The phytoplasma effector TENGU (tengu-su inducer), identified in the onion yellows phytoplasma, induces phenotypes such as stunting, witches’ broom, and sterility. These phenotypes were observed when the effector was transiently expressed in tobacco and Arabidopsis plants. TENGU suppresses auxin response factor 6 (ARF6) and ARF8 genes, disrupting auxin and jasmonic acid (JA) signaling pathways, which leads to the development of these phenotypes (**Figure 2d**). ARF6 and ARF8 are crucial for regulating flower development through the jasmonate pathway (Hoshi et al., 2009; Minato et al., 2014; Tomkins et al., 2018).

### 3.8 SWP1 produced by *Ca.* P. tritici

Another effector produced by *Ca.* P. tritici, also known as wheat blue dwarf phytoplasma, is SWP1 (SAP11-like protein secreted WBD protein 1). This effector interacts with and degrades TCP18 (BRC1), resulting in witches’ broom symptoms in infected.

*N. benthamiana* (Wang et al., 2018a, Wang et al., 2018b). The BRC1 gene mainly functions to halt bud development and encourage vegetative growth and branching. Its degradation hampers vegetative growth, leading to witches’ broom symptoms in infected wheat plants and aiding phytoplasma adaptation within the host.

### 3.9 Secreted ROLP protein 1

**Figure 5:**
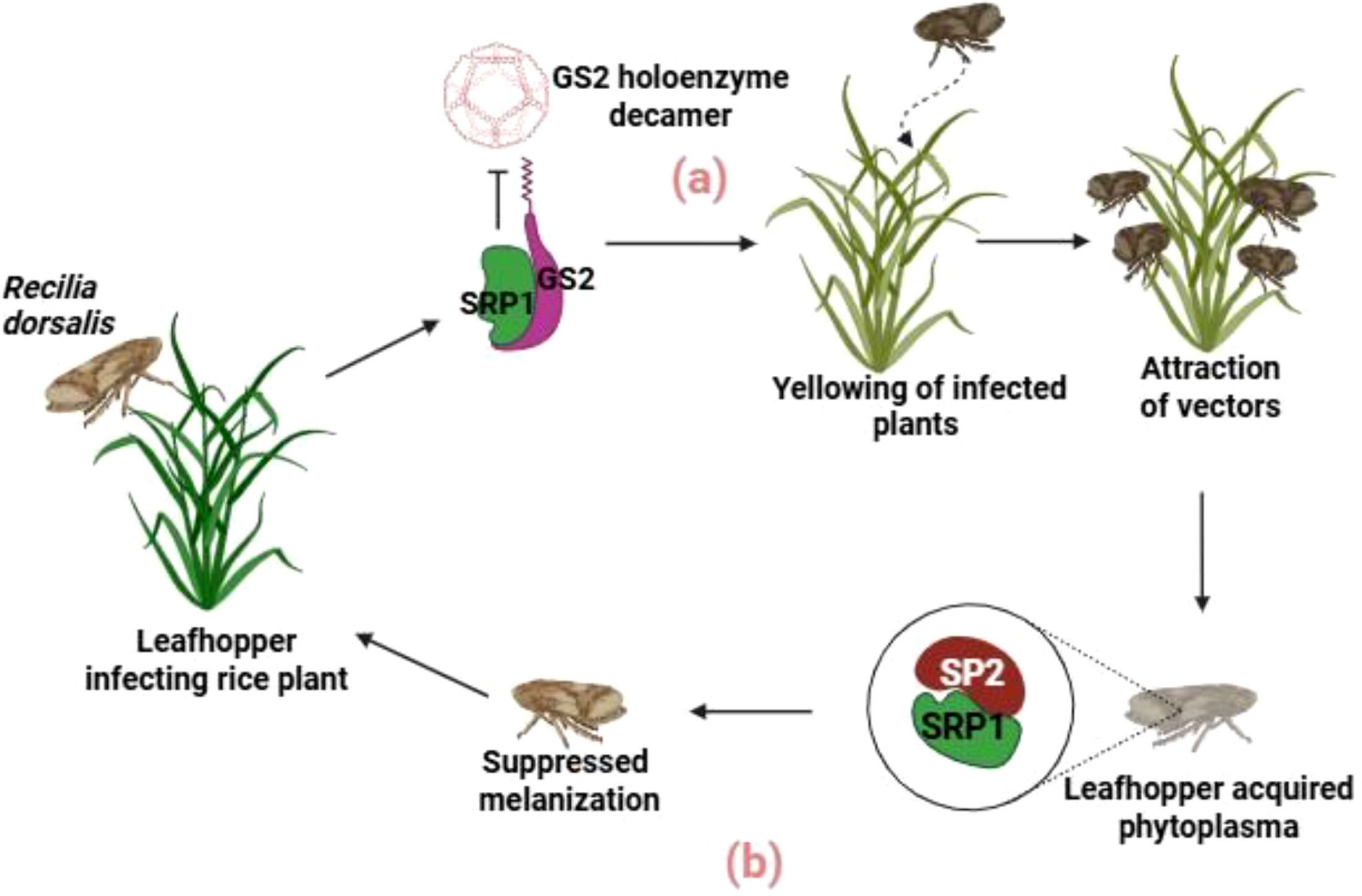
Interface effects between ROLP-rice-zigzag leafhopper. **(a)** SRP1 inhibits the formation of the GS2 holoenzyme decamer by interacting with GS2. This process affects chlorophyll synthesis, causing yellowing symptoms that attract more leafhopper vectors. **(b)** Inside the vector, SRP1 binds to and degrades SP2, resulting in suppressed melanization, an innate immune response in vectors. This image was created using https://BioRender.com.

Rice orange leaf phytoplasma (ROLP) secretes an effector protein called SRP1, which causes leaf yellowing in rice. SRP1 interacts with chloroplastic glutamine synthetase (GS2) and disrupts the formation of the GS2 holoenzyme decamer, which is essential for producing chlorophyll precursors. This disruption reduces the production of glutamate and glutamine, leading to decreased chlorophyll accumulation and the yellowing phenotype. Additionally, these leaf changes attract more leafhopper vectors, increasing ROLP transmission (**Figure 5a**). Therefore, the SRP1 effector manipulates leaf color to enhance the pathogen’s spread (Zhang et al., 2024).

Previously, phytoplasma effectors were known to manipulate insect behavior, increasing their attraction to both healthy and infected hosts. However, the specifics of how these effectors interact with insects were not well-understood. Recently, a groundbreaking discovery showed that the ROLP effector SRP1 inhibits insect melanization, an essential innate immune response. SRP1 binds to serine protease 2 (SP2) and prevents SP2-driven melanization. This defense process involves converting prophenoloxidase to active phenoloxidase via serine proteases, leading to melanization. Experiments demonstrated that microinjecting SRP1 into the ROLP vector, *Recilia dorsalis*, suppressed melanization (**Figure 5b**). This finding provides important insights into phytoplasma-vector interactions (Zhang et al., 2025b). This finding provides important insights into phytoplasma-vector interactions leading to extended phenotypes in infected plants.

These insights indicate that phytoplasma effectors influence the host symptom development by disrupting specific host phytohormones signalling pathways. For instance, SAP11, SJP39, and TENGU target JA, GA and auxin signalling pathways, respectively. Specifically, all the phytoplasma effectors suppress the host gene expression by regulating the genes positively or negatively, which leads to symptom expression. Such molecular interference results in phenotypic modifications mediated by SAP54, SAP11, SAP05 and SRP1 effectors, enhance insect vector attraction to the host. Collectively, this suggests a dual strategy for both systemic colonisation and dissemination through the insect vectors. These mechanisms primarily under the influence of phytoplasma effectors ensure pathogen fitness and transmission.

Understanding the structural domains and motifs of these effectors is therefore crucial for elucidating how they interact with host targets. Moreover, exploring how plants recognize phytoplasma invasion through pattern-recognition receptors and how these interactions can be exploited using modern strategies such as CRISPR-based genome editing and microRNA-mediated regulation may provide new avenues for effective disease management.

## 4 Domains and motifs of phytoplasma effectors and their significance

So far, 717 candidate and 21 validated effectors of phytoplasma have been identified, though not all candidate effectors have been tested for their ability to induce symptoms. Only 153 of these effectors contain characterized functional domains (Carreón-Anguiano et al., 2023). To explore their role in symptom development, the domains and motifs of these effectors were analyzed. Common domains include the sequence variable mosaic (SVM) domain, the TCP binding domain, the coiled-coil (CC) domain, as well as smaller domains such as the ryanodine receptor, collagen triple helix repeat, nucleic acid binding, peptidase, and N-terminal sigma factor regulator domains. Motifs in phytoplasma effectors include eukaryotic linear motifs (ELMs), short linear motifs (SLiMs), coiled-coil motifs, and several unknown motifs. Based on these features, effectors were grouped into 15 families or tribes. A total of 42 ELMs/SLiMs were identified, which may serve as new tools for understanding the molecular mechanisms through which effectors promote infection (Carreón-Anguiano et al., 2023).

The SVM domain found in many effector proteins, plays an important role in shaping the genome organization and effector diversity of phytoplasmas.SVM domains have been identified in 87 phytoplasma effectors, including those under review such as SAP11, SAP54, and SAP05 (Carreón-Anguiano et al., 2023). Functional analyses of SAP11 have demonstrated the importance of specific domains in host manipulation. Mutants of SAP11 lacking the N-terminal domain can still interact with TCPs but cannot destabilize them, while mutants missing the C-terminal domain fail in both binding and destabilizing TCPs. Neither mutant can produce the typical symptoms (Sugio et al., 2014). The key TCP-binding region is within SAP11, which degrades CIN-TCP transcription factors, causing witches’ broom symptoms in infected plants (Pecher et al., 2019). The C-terminal coiled coil domain (amino acids 91–106) is crucial for binding and destabilizing TCPs (Singh et al., 2019). In SAP11 homologs SJP1 and SJP2, the N-terminal and C-terminal CC domains are essential for TCP binding (Zhou et al., 2021). Overall, the N-terminal, C-terminal, and CC domains of SAP11 are vital for interfering with TCPs; deleting any of these domains impairs the degradation process and associated symptom development (Bai et al., 2023).

The 11 amino acids at the N-terminus of the TENGU effector form the conserved functional domain, which alone can induce disease symptoms in the infected plant (Sugawara et al., 2013). The SWP1 effector has a functional C-terminal CC domain; deleting this domain prevents it from inhibiting BRC1 degradation (Wang et al., 2018b). The Zaofeng3 effector is predicted to contain three complete α-helix domains crucial for symptom development. Further research confirmed that all three domains are necessary for the effector’s activity (Chen et al., 2024a).

As discussed above, the PHYL1 and SAP54 effectors promote phyllody by degrading the MADS-box domain. Specifically, PHYL1_PnWB_ interacts with the keratin-like domain of the MADS TF SEP3-K. The crystal structure of PHYL1 shows an α-helical hairpin crucial for binding to the k-domain of SEP3 (Liao et al., 2019). Likewise, SAP54 binds to the keratin-like domain of the MIKC-type MADS-domain family and some RAD23 proteins through the ubiquitin-proteasome pathway, folding into an α-helix. Research has demonstrated that inserting mutations that break the α-helix prevents SAP54 from folding and binding properly to its targets, thus impairing its ability to cause symptoms (Aurin et al., 2020).

Domains are conserved functional units found in most proteins (Vogel et al., 2004). Because of their conserved nature, even a single amino acid change can disrupt their function. For example, deleting the C-terminal domain in SAP11 led to milder symptoms (Bai et al., 2023), which in turn downregulated signaling pathways. This could alter vector attraction, either repelling *M. quadrilineatus* or attracting other vectors, and such outcomes are unpredictable. Additionally, phytoplasma effectors are susceptible to gene loss (Carreón-Anguiano et al., 2023), which may lead to further evolution. Based on this evidence, it is suggested that the loss of critical amino acids through evolution or mutations could impair these domains, reducing the phytoplasma effectors’ ability to induce symptoms. This might challenge the current EP concept of phytoplasma effectors. Consequently, instead of removing the entire effector binding domain, targeting one or two conserved amino acids within these domains could be a more effective strategy for developing complete resistance against phytoplasmas in crops.

## 5 Ecological significance of EP of phytoplasmas

EPs are generally heritable (Whitham et al., 2003). In phytoplasmas, effector genes are thought to be transferred to offspring via horizontal gene transfer, although the mechanisms and evolutionary implications of this process remain unclear (Sugio et al., 2011b). EPs can affect community genetics by influencing not only the host but also other organisms within the community or ecosystem. As a natural part of evolution, genes are products of evolutionary processes, and phytoplasma effectors may have become part of the genome through evolution, adding to genetic diversity within the species (Hogenhout et al., 2008). This diversity causes various effects within the community, forming the basis of community genetics. These effects include interspecific interactions among species, which are heritable because the genetic variation involved is passed down following standard inheritance patterns (Mitton, 2003).

The EPs of phytoplasmas arise from the interaction between the phytoplasma genome and the plant genome during infection, which also triggers the attraction of insect vectors. This linkage can potentially facilitate the pathogen’s spread or attract new insect vectors. Over time, factors like mutation may allow the pathogen to adapt to new insect vectors for transmission (Gray and Banerjee, 1999), influencing the genomes of these vectors. Consequently, these vectors may further evolve into carriers of the pathogen. This process could explain the shift in vector diversity, from an initial primary leafhopper vector to a broader range of insect vectors.

Conversely, if conditions are suitable, the insect vector might begin feeding on the new host and transmit the pathogen, leading to the establishment of new plant hosts. Additionally, the significant vegetative growth of plants during phytoplasma infection attracts secondary pests and pathogens that are not originally present (Costes et al., 2013). This sequence of events initiated by EPs could alter the community genetics within the ecosystem. Therefore, EPs are crucial in these processes.

## 6 Pattern and effector triggered immunity and EPs

Plants use a layered defense system to detect and respond to pathogens, involving at least two levels (Tulum and Caglayan, 2023). The first layer involves plasma membrane pattern recognition receptors (PRRs) that identify common microbial signatures such as Damage associated molecular patterns (DAMPs), Microbe associated molecular patterns (MAMPs), or Pathogen associated molecular patterns (PAMPs), initiating Pattern Triggered Immunity (PTI). Some pathogens evade this initial response by secreting virulence effector proteins that suppress it. To overcome this, the second layer of immunity is activated, involving effector-triggered immunity (ETI), effector-triggered susceptibility (ETS), and hypersensitive response (HR), which lead to a defensive reaction (Kourelis and Van Der Hoorn, 2018).

Interestingly, phytoplasmas lack an outer wall. It remains unclear whether their effectors suppress the plant’s defense mechanisms or help them evade immune detection (Sugio et al., 2011b; Tulum and Caglayan, 2023). However, evidence supports the former. Six non-classically secreted proteins (ncSecPs) from *Ca.* P. ziziphi, which causes witches’ broom in jujube, have been identified as HR suppressors that reduce H_2_O_2_ buildup and inhibit Bax- and INF1-triggered HR. Additionally, all these ncSecPs increase the expression of cell death inhibitors in.

*N. benthamiana*, including pathogenesis-related proteins (NbPR-1, NbPR-2, & NbPR-5) and Bax inhibitors (NbBI-1 & NbBI-2). These findings offer insights into how phytoplasmas suppress host immune responses (Gao et al., 2023). Many modern crop cultivars are vulnerable to phytoplasmas (Bertaccini et al., 2014), supporting the ETI theory mentioned above. Since effectors are crucial for inducing disease symptoms, this may explain the high prevalence of effectors in nature.

## 7 Incorporation of EPs into devising plausible management tools

Managing phytoplasmas involves cultural, physical, mechanical, therapeutic, biological, resistance, and chemical strategies. While insecticides are often recommended for controlling vectors, their effectiveness is sometimes limited (Weintraub and Wilson, 2009). Even with consistent use, their impact is restricted because vectors continuously enter from surrounding areas, and the pathogen spreads quickly. Typically, insecticides can only help lower vector numbers and reduce in-crop transmission (Ustun et al., 2023).

Shoot-tip culture combined with *in vitro* thermotherapy is currently the most promising method for treating phytoplasma infections (Hemmati et al., 2023). However, accurately excising meristems and confirming the elimination of phytoplasmas (Heinrich et al., 2001) remain significant challenges. Alternative approaches, such as using antibiotics, resistance inducers, and various cultural, mechanical, and physical techniques, do not provide robust protection against phytoplasmas. To address these issues, biotechnological tools, especially genome editing (GE), should be advanced to develop durable resistance and ensure complete control over phytoplasmas. Recent studies show that genome editing techniques have potential in managing biotic stresses, including pests and pathogens like insects, fungi, bacteria, and viruses, as well as abiotic stresses (Chen et al., 2024b). Editing susceptible genes, particularly with the progress of CRISPR/Cas systems, has proven highly effective in combating obligate viruses over the past decade, followed by fungal and bacterial pathogens (Boubakri, 2023). These genetic modifications could provide resistance against both phytoplasmas and the insect vectors, offering broad-spectrum protection (Weintraub and Beanland, 2006).

Studies on phytoplasma effectors have uncovered not only how the infection works but also how they suppress plant defenses, as shown in Arabidopsis (Sugio et al., 2011a). Arabidopsis RAD23 mutants, designed to block the SAP54 effector, did not develop phyllody symptoms (MacLean et al., 2011). Since phytoplasmas undergo phase variation across two eukaryotic hosts, targeted studies could lead to methods for controlling their spread (Sugio et al., 2011b). Looking ahead, CRISPR-Cas technology might be used to modify effector binding domains, providing long-lasting resistance to phytoplasmas. At the same time, managing insect vectors is crucial. Genetic engineering can alter insect behavior by editing key genes (Bisht et al., 2019) or making insects less likely to feed.

The characterization of effectors and their mutants has also been reported to enhance resistance against insect vectors.

**Figure 6:**
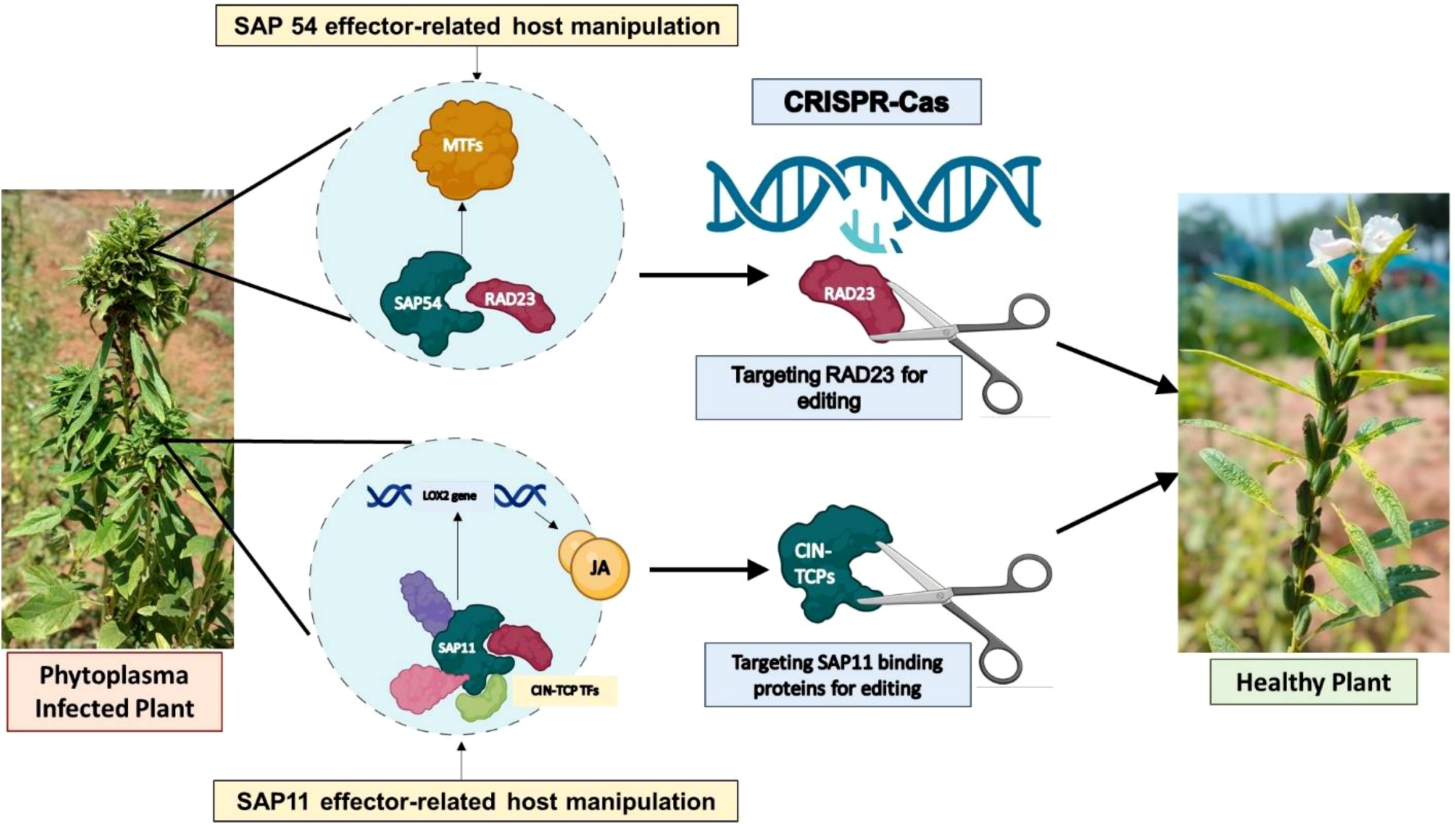
CRISPR-Cas mediated genome editing for phytoplasma management. The proteins SAP54 and SAP11, which are targeted in plants, can be edited with CRISPR-Cas to produce a healthy plant line free of phytoplasmas. This image was created in https://BioRender.com.

In light of that, recently, CRISPR/Cas9 technology was used to knockout the SAP54 target genes in tomato, specifically SlRAD23C and SlRAD23D proteins, to develop resistance to the potato purple top phytoplasma. PCR screening and sequencing confirmed targeted gene deletions in the mutant tomato lines, which will be further studied for their resistance to the disease (Inaba et al., 2025). The MTF mutants with reduced SAP54 activity did not attract insects, whereas infected plants attracted more insects and showed improved insect reproduction and survival (MacLean et al., 2014; Tomkins et al., 2018). This highlights the potential of genome-editing strategies to disrupt SAP54–MTF interactions and thereby reduce vector-mediated phytoplasma transmission. Likewise, knocking out CIN-TCPs to suppress SAP11 expression could potentially decrease insect survival (**Figure 6**). However, these genes regulate key developmental and defense pathways and their modification may lead to pleotropic effects. For example, TCP transcription factors regulate plant architecture and jasmonic acid–mediated defense responses, while SPL genes control phase transition and flowering time; therefore, editing these genes may alter plant growth or reproductive traits (Sugio et al., 2011a; Huang et al., 2021). Also, RAD23 proteins participate in the ubiquitin–proteasome degradation pathway, which influences multiple cellular processes, so disrupting this pathway may affect stress responses or overall plant vigor (Huang et al., 2021). While genome editing may enhance resistance to phytoplasma diseases, breeders must carefully evaluate potential impacts on yield stability, plant architecture, stress tolerance, and crop management practices, since the targets of phytoplasma are important in plant growth and development, ensuring that resistant traits do not compromise agronomic performance. In addition, differences in regulatory frameworks for genome-edited crops create legal and implementation challenges, and strategies such as vector genome editing require careful ecological risk assessment due to possible impacts on insect populations and ecosystem balance.

MicroRNAs (MIRs) are crucial post-transcriptional gene regulators in plants, playing vital roles in plant immunity by modulating key hormones and controlling defense-related genes (Luo et al., 2024). They also influence normal growth processes such as stunting, dwarfing, flower development, and shoot and root growth. The phytoplasma infection considerably modulates the miRNA levels in crop plants. Evidence suggests that *Ca.* P. asteris-infected *Catharanthus roseus* exhibited upregulation and downregulation of several small RNAs. The infected plants exhibited virescence (vir) and witches’ broom (WB) plants and both the plants regulated different levels of miRNA. For instance, miR156 and miR319 was slightly increased in virescence plants and slightly decreased in witches’ broom plants; conversely, miR157 was downregulated in vir plants and upregulated in WB plants. Other miRNAs shows similar response regardless of symptoms produced: miR159 and miR162 were consistently downregulated in both; miR166 and miR172 levels were increased in the infected plants and miRNAs such as, miR167, miR168, miR170, miR171, miR390, miR391 and miR396 showed decrease expression in the infected plants (Contaldo et al., 2023). This differential miRNA regulation in virescence and witches’ broom exhibiting plants may be correlated with the production of specific effectors by the phytoplasmas since effectors are responsible for the production of different symptoms.

Research shows that phytoplasma infection produces effectors that impact MIR levels, primarily regulating three MIRs: miR156, miR172, and miR159 (Kumari et al., 2023). miR156 and miR172 are major regulators of flowering time, targeting SPL and AP2 genes involved in the EP mechanism affected by effectors (Teotia and Tang, 2015). The SAP54 effector modifies miR156 levels, with overexpression leading to smaller leaves (Zhang et al., 2011), extended vegetative growth, and delayed flowering (Wang et al., 2009). During phytoplasma infection, both SAP54 and PHYL1_PnWB_ suppress miR156h, miR159, miR390, and miR396. PHYL1_PnWB_ increases the MADS-box TF SVP and decreases miR396, causing leafy flower traits in Arabidopsis. However, how PHYL1 affects AtSVP and miR396 is still unclear, and there is no evidence that miR396 mediates AtSVP degradation (Yang et al., 2015).

SAP11 effector expression also increases miR156 levels, although the precise mechanism remains unclear (Chang et al., 2018; Kumari et al., 2023). The SAP11 effector secreted by jujube witches’ broom was also found to raise zju-miR156c levels, with ZjSPL3 being its target gene (Wang et al., 2024b). Additionally, miR157 was observed to elevate during peanut witches’ broom infection significantly (Ehya et al., 2013). Overexpressing this miRNA in *Torenia fournieri* led to notable phenotypic changes, such as increased branching and smaller leaves (Shikata et al., 2012). Specifically, this miRNA regulates SPL protein expression, which is involved in the SAP05 effector mechanism. Overall, these findings suggest that miR157 is crucial in altering plant architecture and morphology, the extended phenotypes driven by phytoplasma effectors (Ehya et al., 2013).

MIRs are essential in plant development, but further research is needed to understand the molecular mechanisms controlling MIRs. Significant progress includes studying how miRNAs interact with components of flowering pathways and the upstream processes in miRNA biogenesis influenced by phytoplasma effectors (Kumari et al., 2023). To fully understand and utilize miRNAs, identifying their target genes is crucial, as this could help decode the intricate miRNA-regulated networks that govern plant responses to both biotic and abiotic stresses (Ehya et al., 2013). Ultimately, this miRNA framework offers a valuable basis for understanding symptom formation and developing strategies for disease management (Lakhanpaul et al., 2023).

## 8 Conclusion

EPs are a fascinating natural phenomenon increasingly significant today. They are environmental adaptations of parasites, shaped by evolution and natural selection to enhance their reproduction and survival. This reflects Darwin’s idea of ‘from simple beginnings to complex adaptations.’ In parasitic EPs, organisms devoid of nervous systems have evolved advanced strategies to manipulate host phenotypes. Although host manipulation provides significant advantage to parasites, it entails a substantial energetic cost. Consequently, parasite-driven host control represents a remarkable adaptation with ecological and evolutionary impacts, both positive and negative, warranting further study.

Understanding the mechanistic framework underlying these extended phenotypes is essential for clarifying their role in natural ecosystems. Particularly, in plant-microbe interactions, plant microbial communities influence the plants positively by improving plant traits, including resistance to various stresses. Whereas, the pathogen-derived effectors manipulate the host morphology and physiology to facilitate their infection and transmission. Insights on these mechanisms provide a conceptual bridge between biological understanding and practical agricultural applications. Accordingly, future research should explore how these mechanisms can be leveraged to suppress plant pathogens. Building on these principles, strategies can be devised to target and inhibit harmful phytoplasmas that produce effectors. Moving away from traditional management methods that rely heavily on harmful insecticides, innovative biotechnologies such as genome editing may offer safer and more sustainable alternatives. Experiments have shown that silencing effectors through targeted mutation of key regulators leads to long-lasting pathogen resistance, providing an effective way to combat phytoplasma associated diseases. Overall, EPs not only deepens our knowledge of host-parasite evolution but also offers great potential for boosting host resistance to pathogens and advancing sustainable plant health management.
