# Supplementary material for: Extended phenotype of phytoplasmas in eukaryotic systems: mechanisms and ecological implications
Source: Front Plant Sci. 2026 Apr 10;17:1774394. doi: 10.3389/fpls.2026.1774394 (PMC13106440; doi:10.3389/fpls.2026.1774394)
Supplement: Supplementary file 1 [file Table1.docx]

**Table S1. Evidences of extended phenotypes in fossils**

| **Specimen** | **Site / Location** | **Parasite** | **Host** | **Resultant phenotype** | **Beneficial / harmful** | **References** |
| --- | --- | --- | --- | --- | --- | --- |
| Halloween pumpkin masks | Marine ecosystem | Copepods | Corals | Results in cyst formation | Harmful to host | Boucot and Poinar Jr (2010) |
| 47 Myr old fossil leaf with scars | Messel deposits of Germany | *Ophiocordyceps unilateralis* | Carpenter ants (*Camponotus leonardi*) | Zombie ants and death grip behaviour | Harmful to host | Hughes et al. (2011) |
| 23 Myr old Rhizocephala  (Barnacles infecting crabs) | New Zealand | Crab hacker barnacle (*Succulina carcini*) | Crabs (*Tumidocarcinus giganteus*) | Infected male hosts feminized and brood the parasitic offspring | Harmful to host | Feldmann (1998) |
| Suicidal insects | - | Mermithids & hairworms | Crickets | Suicidal drowning of insects due to manipulation of maturing parasites | Harmful to host | Boucot and Poinar Jr (2010) |
| Scale insects from cretaceous period | - | *Paleoophiocordyceps coccophagus* | Scale insects | Ability of parasites to correctly identify the hosts | Harmful to host | Sung et al. (2008) |
| Worker ants with punctured ants | - | Tetradonematid nematode (*Myrmeconema antiqua*) | Worker ants (*Cephalotes serratus*) | Gasters of ants turns red inviting the birds to pick it up and disseminate the parasites dwell in the gaster | Harmful to host | Poinar (2011) |
| Burmese amber termite | - | Rhabditidae nematodes | Termites | Predators attack termites leading to dissemination of parasites | Harmful to host | Poinar (2011) |

**Table S2. Example of extended phenotypes**

| **S. No.** | **Parasite / organism** | **Host** | **Vector** | **Extended phenotype** | **Beneficial / Harmful** | **References** |
| --- | --- | --- | --- | --- | --- | --- |
| **Animal hosts** | | | | | | |
|  | *Ophiocordyceps unilateralis* fungus | Carpenter ants (*Camponotus leonardi*) | Spores of parasite spread through air | Death grip / lock jaw behaviour of zombie ants | Harmful to host | De Bekker et al. (2015) |
|  | *Plasmodium falciparum* (Protozoan) | Humans | *Anopheles gambiae* (Mosquito) | Mosquitos manipulated to bite more humans | Harmful to host | Smallegange et al. (2013) |
|  | Cuckoo | Large-billed Gerygone | - | Brood parasitism | Harmful to host | Sato et al. (2010) |
|  | *Ribeiroia* trematodes | Frog | Birds | Severe limb deformities on frog | Harmful to host | Johnson et al. (2004) |
|  | *Spinochordodes tellinii* (Nematodomorpha) | Crickets | Crickets | Suicidal drowning | Harmful to host | Libersat et al. (2009) |
|  | - | Termites | - | Termite mounds and fungal gardens | Beneficial to host and environment | Enagbonma and Babalola (2019) |
|  | Iridovirus | Crickets | Crickets | Fatal attraction - infected males attracted to healthy females | Harmful to host | Adamo (2014) |
|  | *Myrmeconema antiqua* (Nematode) | Giant turtle ant (*C. atratus*) | Birds | Cherry red ant gasters | Harmful to host | Hubregtse (2019) |
|  | *Toxoplasma gondii* (Protozoa) | Leopard | Chimpanzee | Fatal attraction of chimpanzees to tigers | Harmful to host and vector | Poirotte et al. (2016) |
|  | Algae | Corals | - | Coral reefs | Beneficial symbiosis | Parkinson and Baums (2014) |
|  | *Toxoplasma gondii* (Protozoa) | Mice | Cats | Fatal attraction of mice to cats | Harmful to host | Vyas et al. (2007) |
|  | *Neoleucochloridum problematicum* (Trematode) | Snails | Birds | Loss of movement to get eaten by predatory birds | Harmful to host | Kagan (1952) |
|  | Hz-2V virus | *Helicoverpa zea* (Butterfly moth) | Male moths | Infected female produces 5 to 7 times more sex phermones | Harmful to host | Burand et al. (2005) |
| **Plant hosts** | | | | | | |
|  | Gall wasps | Oak trees | - | Galls on oak trees | Harmful to host | Bailey et al. (2009) |
|  | Tomato spotted wilt virus (TSWV) | Tomato | *Frankliniella occidentalis* | Host pathways manipulated  Increase in reproduction and survival of vector | Harmful to host | Nachappa et al. (2020) |
|  | Barley yellow dwarf virus (BYDV) | Barley | *Rhopalosiphum padi* | Infected vectors prefer healthy hosts, healthy vectors prefer infected hosts | Harmful to host | Ingwell et al. (2012) |
|  | Tomato yellow leaf curl virus (TYLCV) | Tomato | *Bemisia tabaci* (Whitefly) |  |  | Fang et al. (2013) |
|  | *Candidatus* Liberibacter asiaticus | *Citrus* sp. | *Diaphorina citrii* (Psyllid) |  |  | Mann et al. (2012) |
|  | *Puccinia and Uromyces* | *Wide host range* | *Pollinating insects* | Pathogens produce pseudoflowers to mimic the real flowers and attracts insects for transmission | Harmful to host | Ngugi & Scherm, 2006 |
|  | Downy mildew pathogen | Graminae plants | Wind and insect | Witches broom exposed spores for dispersal | Harmful to host | Thines et al. (2008) |
|  | Black raspberry necrosis virus (BRNV)  Raspberry leaf mottle virus (RLMV) | Red raspberry | Raspberry aphid, *Amphorophora idaei* | Delayed maturity of insect vectors feeding on infected plants | Harmful to host | McMenemy et al. (2012) |
|  | *Rhodococcus fascians* (Actinomycete) | Wide range of crops | - | Galls produced by producing excess cytokinins | Harmful to host | Giron and Glevarec (2014) |
|  | *Pseudomonas syringae* | Broad host range | Wide range of vectors | Modulates apoplastic composition  Interferes with signalling pathways  Attracts insects | Harmful to host | Ma and Ma (2016) |
|  | *Sporisorium reilianum* | Maize | - | Partial or complete phyllody of inflorescence | Harmful to host | Ghareeb et al. (2019) |
|  | *Agrobacterium tumefaciens* | Broad host range | - | Formation of crown galls (tumours) | Harmful to host | Mashiguchi et al. (2019) |
|  | Fungal endophytes | Plants | - | Endophytes helps plants by enhancing biotic and abiotic stress resistance | Beneficial to host and microbes | Hawkes et al. (2021) |
|  | Plants | Soil | - | Improves physicochemical properties and rhizodeposition recruits diverse microbes | Beneficial to plants, soil and microbes | de La Fuente Cantó et al. (2020) |
